# Optimizing the pathology workstation “cockpit”: Challenges and solutions

**DOI:** 10.4103/2153-3539.70708

**Published:** 2010-10-01

**Authors:** Elizabeth A. Krupinski

**Affiliations:** Department of Radiology, University of Arizona, Tucson, AZ 85724, USA

**Keywords:** Cockpit, digital dashboard, informatics

## Abstract

The 21^st^ century has brought numerous changes to the clinical reading (i.e., image or virtual pathology slide interpretation) environment of pathologists and it will continue to change even more dramatically as information and communication technologies (ICTs) become more widespread in the integrated healthcare enterprise. The extent to which these changes impact the practicing pathologist differ as a function of the technology under consideration, but digital “virtual slides” and the viewing of images on computer monitors instead of glass slides through a microscope clearly represents a significant change in the way that pathologists extract information from these images and render diagnostic decisions. One of the major challenges facing pathologists in this new era is how to best optimize the pathology workstation, the reading environment and the new and varied types of information available in order to ensure efficient and accurate processing of this information. Although workstations can be stand-alone units with images imported via external storage devices, this scenario is becoming less common as pathology departments connect to information highways within their hospitals and to external sites. Picture Archiving and Communications systems are no longer confined to radiology departments but are serving the entire integrated healthcare enterprise, including pathology. In radiology, the workstation is often referred to as the “cockpit” with a “digital dashboard” and the reading room as the “control room.” Although pathology has yet to “go digital” to the extent that radiology has, lessons derived from radiology reading “cockpits” can be quite valuable in setting up the digital pathology reading room. In this article, we describe the concept of the digital dashboard and provide some recent examples of informatics-based applications that have been shown to improve the workflow and quality in digital reading environments.

## INTRODUCTION

Digital pathology is attracting more and more converts, with new companies getting into the market and more pathologists realizing the potential benefits of digital imaging for improving patient care, improving workflow, contributing to personalized medicine and advancing pharmaceutical research.[1–3] As more pathologists interact with these digital systems, however, it is becoming clear that the transition from glass slides and light-based microscopy may not be as smooth as one would hope. There are a number of benefits to be gained with digital pathology, but there are also a significant number of challenges yet to be overcome and some yet to be encountered.[1] One of these challenges is the optimization of the workstation “cockpit.” As in radiology, the pathologist and the workstation can be compared to a pilot and a plane. Clearly, there needs to be solid structural foundation for either to function properly and efficiently – the plane for the pilot and the physical workstation and display for the pathologist.

Based on years of experience in radiology and the transition from film to digital imaging, we already have a great deal of knowledge about how to optimize the medical imaging workstation and reading environment, and many of these lessons can be directly applied to pathology virtual slide viewing.[4] In radiology, the first real discussions about “going digital” and the digital reading environment started to occur in the late 1970s and the early 1980s.[5,6] The term Picture Archiving and Communications Systems (PACS) was coined at the First International Conference and Workshop on Picture Archiving and Communication Systems held in Newport Beach, CA, in January 1982 and similar efforts occurred in Asia and Europe during the same time frame. Practical deployments occurred during the 1990s. A worldwide survey in 1998[7] revealed that there were 177 PACS in operation. There are no real records of how many departments progressively went digital vs. those that went “cold turkey,” but, today, PACS and digital radiography have penetrated nearly every market worldwide, including other radiographic specialties such as dentistry, cardiology and veterinary radiology.

Some of the key factors, in addition to hundreds of supporting studies, that contributed to the success of PACS included the introduction and acceptance by both the clinical and the manufacturing communities of image and data format standards (DICOM or the Digital Imaging and Communications in Medicine Standard), the evolution of informatics and key enabling technologies such as laser film digitizers, computed radiography, digital storage units, advanced medical grade computer displays and asynchronous transfer mode (ATM) network capabilities.[8] Although most PACS systems enable other clinical specialties to access radiology images and report data, the adoption of PACS and integration into other specialties has been rather slow. In part, this is because DICOM has been only slowly adapted to the other image-based specialties (especially those that involve color images since color calibration is much more difficult than grayscale), but the core PACS technologies rely on DICOM. Vendors are highly invested in DICOM and the idea of developing and converting to some other standard format is not very attractive.

One of the main differences of course is that pathology workstations have the added complexity of using color displays and the associated challenges of how to calibrate them properly.[9,10] An even greater challenge, however, even in radiology, is optimizing the management and monitoring of the massive amounts of information now available in electronic forms in hospitals as it flows between and within departments and healthcare facilities. This is where informatics and the digital dashboard or cockpit comes into play.

## WHAT IS INFORMATICS?

Informatics “is the discipline focused on the acquisition, storage and use of information in a specific setting or domain.”[11] The digital dashboard as an informatics tool can help facilitate the efficient *use* of image information by clinicians as they *visualize* and accurately *interpret* medical images. The digital dashboard is a portal to information as well as an active miner and integrator of information. It can be designed to integrate separate computerized information systems (e.g., Hospital Information System [HIS] and other clinical information systems) and summarize key work flow metrics in real time to facilitate informed decision making. In radiology, digital dashboards have been designed to alert radiologists to their unsigned report queue status, facilitate the transcription process by providing report templates, provide a link to the report signing application and, generally, assess workflow throughout the chain from image acquisition to reporting.[12–17] Digital dashboards have, in some cases, been shown to significantly improve workflow[12] and potentially reduce image retakes (i.e., reduce excess dose to patients) by tracking technologist use patterns.[16]

## DIGITAL DASHBOARDS

Although there are a number of digital dashboard concept designs, in-house products that have been developed and used and companies that have started to offer digital dashboards with their products, what is surprising and sometimes disconcerting is that there are really no standards or guidelines with respect to what metrics are important and what exactly should be measured and monitored with the digital dashboard. The Health Information Technology for Economic and Clinical Health Act may help in this regard as one of its byproducts is the Meaningful Use legislation, which incorporates incentives for using health information technology that incorporates means for establishing and reporting quality measures, but even these efforts do little to help define what these measures are and what metrics should be used.[18,19] Pathology professional societies and, in particular, those involved with informatics should actively pursue definitions of meaningful use in pathology and establish the metrics and benchmarks for reporting of quality performance. Once these are formulated, informaticists can develop the necessary tools, within the context of the digital dashboard, for monitoring and reporting these metrics and outcomes.

Digital dashboards arose well after digital workstations and PACS proliferated in radiology and, thus, at least initially, were designed in an ad hoc fashion rather than in a prospective manner. This is where pathology has a potential advantage over radiology. Pathology informaticists can be proactive – designing digital dashboards now, when the digital reading environment is still in its early development and implementation. Many of the tasks that the radiology digital dashboards are designed to impact can be found in pathology as well. The opportunity to proactively design digital dashboard applications and tools and incorporate them into the growing numbers of pathology information systems and workstations is ripe.

For example, improving report turnaround times (including generation and signing) is a goal in many clinical specialties.[20,21] Morgan *et al*. developed an unsigned report monitor for incorporation into their digital dashboard and examined its influence on the report turnaround time of radiologists. The application used the traffic light metaphor in which red, yellow and green circles were used in conjunction with the image queue to indicate when there were more than 30 (red) unsigned reports, between 1 and 20 unsigned reports (yellow) and no unsigned reports (green). When a red or yellow alert appears, the radiologist simply clicks on it and the appropriate program (electronic signature application in this case) to remedy the problem is automatically launched. With this very simple alert system and user interface, they observed a 24% reduction in time between transcription and report finalization from about 24 h to about 18 h.[12]

A more recent study examined the further impact of adding financial incentives to information technology.[22] They implemented three interventions to improve the report signature component of the total report turnaround time. The first two were technology-based: a notification paging application alerting radiologists when reports were ready to be signed and a PACS-integrated speech recognition report generation system. The third component was a departmental financial reward (semiannually) for signing performance. The technology alone significantly reduced signature times and the addition of the financial incentives reduced it even further.

Adapting informatics tools such as these to the digital pathology reading environment can be readily accomplished, with similar positive impacts. Although turnaround times clearly impact patient care by providing more efficient transmission of critical diagnostic information, the quality of that information can be improved using digital dashboard tools as well. Owens *et al*. have developed a pre-sign-out quality assurance informatics tool that randomly accesses a certain percentage of reports for a second pathologist to review before the final report is released.[23] The goal is to record and report disagreements (and levels of disagreement) so that steps can be taken to resolve discrepancies. When fully implemented, it can serve as a prospective tool to prevent errors from occurring, thereby improving patient care.

## SUMMARY

These are just a few examples of how informatics-based applications can be created and incorporated into a digital dashboard for pathology workstations for improving workflow and the quality of patient care. The potential to improve patient outcomes is high as well if the information that the pathologist receives and provides is processed in a more efficient and effective (error-free) manner. Some of the essential features of today’s radiology dashboards include Relative Value Units, unsigned report status, unread studies, average patient wait times, scanner utilization rates and current availability, number and type of cases read in a given time period by an individual, department or facility and automatic distribution of cases to be read as a function of who is logged on the system and how many (and what type) cases each individual is reading (also taking into account their subspecialty).

As virtual slides and other new technologies are adopted, the highest quality pathology services can be assured by proactively designing the future digital pathology reading environment, by incorporating informatics tools to optimize the use of information by the pathologist and by generating standards and metrics for assessing and monitoring performance that these informatics tools can incorporate. One of the challenges for pathology however is the fact that unlike radiology, there are multiple information systems (e.g., anatomical, clinical, molecular) that need to be considered. Radiology departments typically have only a single unified radiology information system (or similar ones that can be easily integrated into the main one such as a separate mammography system). Therefore, the need to integrate different systems has only recently emerged as other specialties have moved to digital reading.

Ideally, of course, one would like to envision entire hospitals and/or healthcare enterprises utilizing the same unified information system and digital dashboard. There are products on the market, but most of them are more directed at business analytics, reporting and management than at clinical data management and analytics. The few enterprise-wide solutions that are available (e.g., Agfa) are still very much image- and report-based systems that do not quite capture the entire enterprise. Developing and agreeing upon the standards that would be required to capture, analyze and manage the immense amount and different types of information generated by a healthcare enterprise may be farther in the future than desirable, but that may be the reality of the situation for now. Although less-unified, the adoption of digital dashboards by individual departments such as pathology that are more similar to radiology than other specialties is likely to be the way of things in the near-future time frame.
